# A Gender-Informed Smoking Cessation App for Women: Protocol for an Acceptability and Feasibility Study

**DOI:** 10.2196/60677

**Published:** 2024-12-10

**Authors:** Osnat C Melamed, Kamna Mehra, Roshni Panda, Nadia Minian, Scott Veldhuizen, Laurie Zawertailo, Leslie Buckley, Marta Maslej, Lorraine Greaves, Andreea C Brabete, Jonathan Rose, Matt Ratto, Peter Selby

**Affiliations:** 1 INTREPID Lab Centre for Addiction and Mental Health Toronto, ON Canada; 2 Department of Family and Community Medicine University of Toronto Toronto, ON Canada; 3 Campbell Family Mental Health Research Institute Centre for Addiction and Mental Health Toronto, ON Canada; 4 Institute of Medical Sciences University of Toronto Toronto, ON Canada; 5 Department of Pharmacology and Toxicology University of Toronto Toronto, ON Canada; 6 Addictions Division Centre for Addiction and Mental Health Toronto, ON Canada; 7 Department of Psychiatry University of Toronto Toronto, ON Canada; 8 Krembil Centre for Neuroinformatics Centre for Addiction and Mental Health Toronto, ON Canada; 9 Centre of Excellence for Women's Health Vancouver, BC Canada; 10 School of Population and Public Health Faculty of Medicine University of British Columbia Vancouver, BC Canada; 11 Edward S. Rogers Sr. Department of Electrical and Computer Engineering University of Toronto Toronto, ON Canada; 12 Faculty of Information University of Toronto Toronto, ON Canada; 13 Schwartz Reisman Institute for Technology and Society University of Toronto Toronto, ON Canada; 14 Dalla Lana School of Public Health University of Toronto Toronto, ON Canada

**Keywords:** smoking cessation, mHealth, co-development, feasibility, smoking, mobile app, cigarette smoking, tobacco cessation, gender-informed design, app design, women's health, behavior change, health behavior change, mobile phone

## Abstract

**Background:**

Tobacco smoking remains the leading preventable cause of death and disease among women. Quitting smoking offers numerous health benefits; however, women tend to have less success than men when attempting to quit. This discrepancy is partly due to sex- and gender-related factors, including the lower effectiveness of smoking cessation medication and the presence of unique motives for smoking and barriers to quitting among women. Despite the gendered nature of smoking, most smoking cessation apps are gender-neutral and fail to address women’s specific needs.

**Objective:**

This study aims to test the acceptability and feasibility of a smartphone app that delivers gender-informed content to support women in quitting smoking.

**Methods:**

We co-developed a smoking cessation app specifically tailored for women, named My Change Plan-Women (MCP-W). This app builds upon our previous gender-neutral app, MCP, by retaining its content grounded in behavioral change techniques aimed at supporting tobacco reduction and cessation. This includes goal setting for quitting, identifying triggers to smoking, creating coping strategies, tracking cigarettes and cravings, and assessing financial savings from quitting smoking. The MCP-W app contains additional gender-informed content that acknowledges barriers to quitting, such as coping with stress, having smokers in one’s social circle, and managing unpleasant emotions. This content is delivered through testimonials and animated videos. This study is a prospective, single-group, mixed methods investigation in which 30 women smokers will trial the app for a period of 28 days. Once participants provide informed consent, they will complete a baseline survey and download the app on their smartphones. After 28 days, participants will complete follow-up surveys. Acceptability will be assessed using the Theoretical Framework of Acceptability, which evaluates whether participants perceive the app as helpful in changing their smoking. The app will be deemed acceptable if the majority of participants rate it as such, and feasible if the majority of the participants use it for at least 7 days. Furthermore, after the 28-day trial period, participants will complete a semistructured interview regarding their experience with the app and suggestions for improvement.

**Results:**

Development of the MCP-W app was completed in September 2023. Participant recruitment for testing of the app commenced in February 2024 and was completed in July 2024. We will analyze the data upon completion of data collection from all 30 participants. We expect to share the results of this acceptability trial in the middle of 2025.

**Conclusions:**

Offering smoking cessation support tailored specifically to address the unique needs of women through a smartphone app represents a novel approach. This study will test whether women who smoke perceive this approach to be acceptable and feasible in their journey toward smoking cessation.

**International Registered Report Identifier (IRRID):**

DERR1-10.2196/60677

## Introduction

Cigarette smoking is the leading cause of morbidity and mortality among women in Western countries through cancer, heart, and lung diseases [1]. The risk for developing smoking-related diseases is higher among women than men, mainly due to their body size and other biological factors [2]. Both men and women share a similar interest in quitting and attempt to quit at similar rates; however, women are 30% less likely than men to quit successfully [3]. This discrepancy can be partly explained by the influence of sex and gender on smoking behavior. Research suggests male brains exhibit a greater pleasurable response to nicotine than female brains [4]. Whereas smoking behavior is propelled by the pleasurable response to nicotine (ie, addiction) among men, it may be propelled by other motives among women, such as external social cues [5]. This partly explains why nicotine replacement therapy is less effective in women compared with men [6]. Women rely on smoking for stress relief to a greater degree than men [7]. Consequently, women in adverse social and economic circumstances, who depend on smoking for stress reduction, encounter particular difficulties in quitting [8]. Furthermore, many women lack support for quitting from their partners, friends, and others in their environment [7]. This hampers women’s efforts in both initiating abstinence and maintaining it [3,9]. Despite the presence of sex- and gender-related influences on smoking, most smoking treatment guidelines are sex- and gender-neutral and do not call for a differentiated approach for women smokers that is based on advances in sex and gender science. Inadvertently, this may perpetuate existing gender disparities in quit outcomes [3,9].

Recently, greater attention has been given to sociocultural contexts that are conducive to smoking initiation and its maintenance and their interaction with the psychopharmacological effects of nicotine [10]. The sociopharmacology theory provides an explanation for the disparity in cessation outcomes between men and women. The theory posits that adverse social circumstances (eg intimate partner violence, lone parenting) make nicotine particularly rewarding and that stress related to these adverse social circumstances then becomes a conditioned cue for cigarette smoking. Since women face more life stressors than men due to societal inequalities, they are less likely to succeed in quitting. It has been suggested that offering behavioral interventions that offset sociocontextual factors in combination with cessation pharmacotherapy could improve quit outcomes for women [11].

Mobile health technologies, such as smartphone apps, can effectively deliver interventions to aid smokers in quitting [12]. They provide an opportunity to expand on existing gender-neutral approaches by addressing sociocontextual factors that influence smoking behavior among women. Apps have a number of advantages compared to traditional in-person interventions, including having a wide reach, being available at all times, and reducing treatment time and travel needs. Also, apps are easily customizable and allow for tailoring of content to meet the needs of specific populations. Indeed, a previous review identified 33 unique smoking cessation smartphone apps with about half targeting specific groups, such as people with mental illness, youth, and pregnant women [13]. Yet, only one app was tailored for nonpregnant women [14]. Armin et al [14] developed a gender-specific app to target multiple unhealthy behaviors among women, including smoking, physical inactivity, and low-quality diet. The app delivered guided imagery to women to reduce their concerns regarding postcessation weight gain and negative body image, which is reported by some women as a barrier for quitting [15]. While fear of postcessation weight gain is a known barrier to cessation among women, there is a need to address the influences of additional sociocontextual barriers in smoking cessation apps for women.

To address this need, we developed a gender-informed evidence-based smoking cessation app with input from patient partners and a multidisciplinary team of clinicians and researchers [15-18]. The app is grounded in the principles of both gender and sociopharmacology theories [10,19]. This theory-based approach allows for an appreciation of the social determinants of smoking behavior in women and appropriate mitigation strategies. This newly developed My Change Plan-Women (MCP-W) app provides women with psychoeducation on the sociocontextual factors that influence smoking behavior, offers strategies to overcome gender-specific barriers to quitting, and provides encouragement through testimonials from women who have quit. In addition, MCP-W contains gender-neutral content such as tracking cigarette use, a diary of urges and cravings, cost savings associated with reduction or cessation, and motivation reinforcement through app notifications. The aim of this mixed methods study is to investigate the acceptability and feasibility of the MCP-W app among women smokers.

## Methods

### Development of MCP-W

Our team has previously developed an evidence-based app for smoking cessation to support the efforts of smokers who enroll in the publicly funded Smoking Treatment for Ontario Patients (STOP) program [20]. The app, My Change Plan (MCP) [21], contains gender-neutral content grounded in behavioral change techniques for supporting tobacco reduction and cessation [22]. This includes the development of a personalized change plan, which allows users to set goals, identify triggers to smoking, create coping strategies, seek support systems, track their cigarettes and cravings, and assess financial savings from reducing or quitting smoking. The app also contains evidence-based content on the different smoking cessation medications, local support for smoking cessations (ie, smokers’ helpline), and general content on how to improve other aspects of health when trying to quit including recommendations for physical activity, nutrition, low-risk alcohol consumption, stress reduction, and sleep hygiene.

Between July 2022 and August 2023, we developed a women-specific version of the MCP app, MCP- W. The MCP-W app was developed in collaboration with patient partners (ie, women with lived experience of smoking) and subject matter experts in tobacco addiction, sex, and gender science, implementation science, public health, app development, and human-computer interactions. We chose to co-design the app with patient partners to ensure the app is relevant to our target population in accordance with the best evidence of co-design principles [23]. We invited 5 patient partner women who were treated in the STOP program for smoking cessation in the past, to co-develop the content of the app.

### Gender-Informed Content in MCP-W

**Figure 1:**
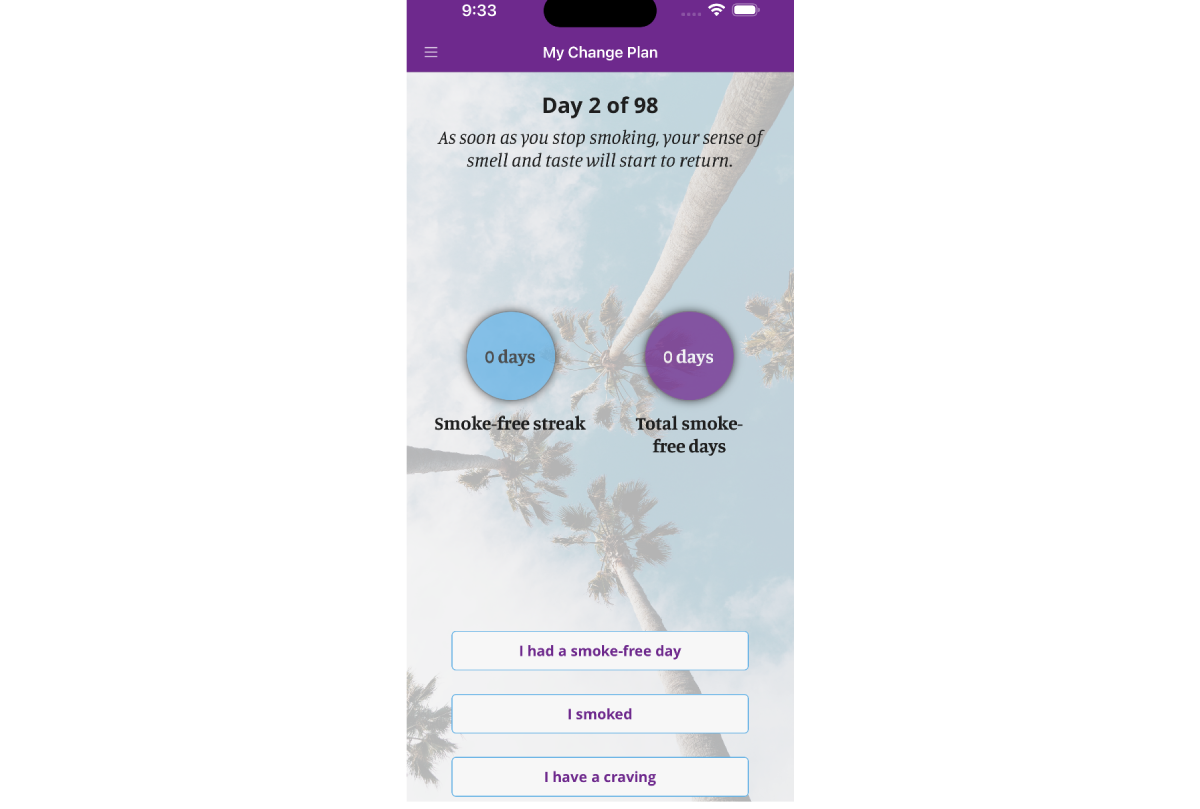
Home page of the My Change Plan-Women app.

**Figure 2:**
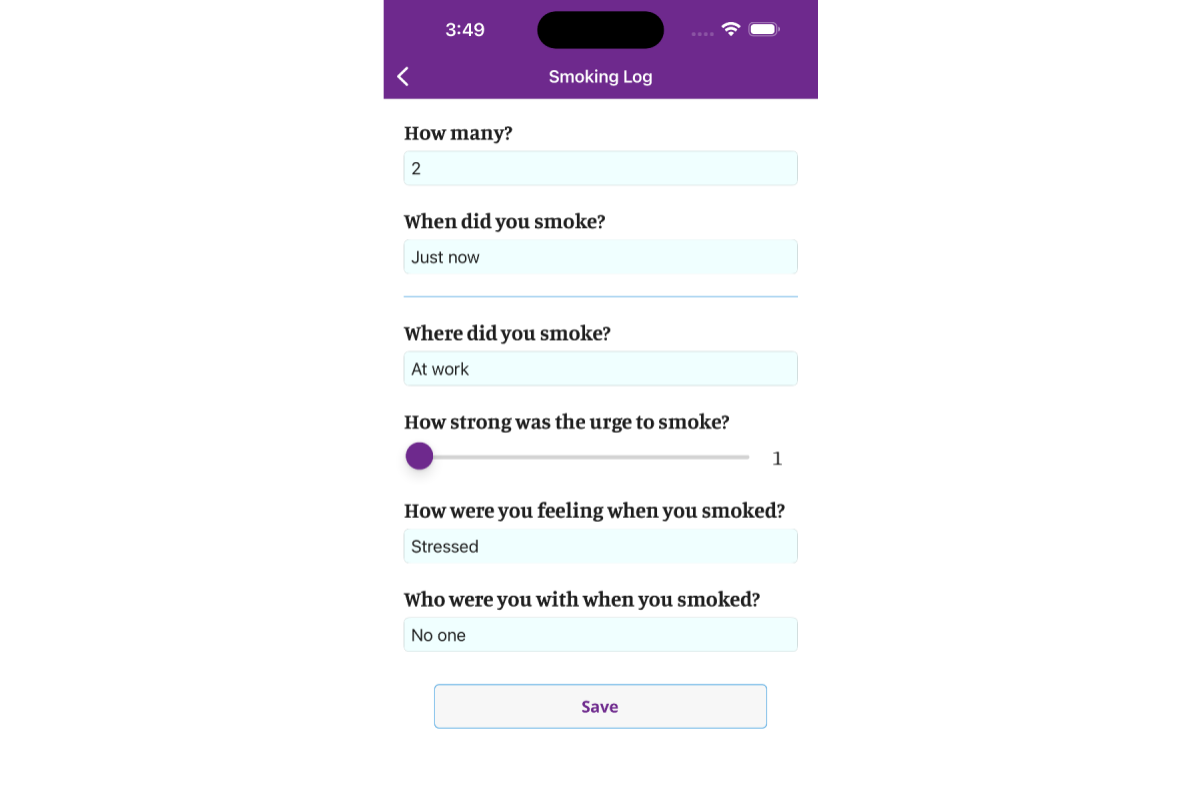
Logging cigarettes in the My Change Plan-Women app.

**Figure 3:**
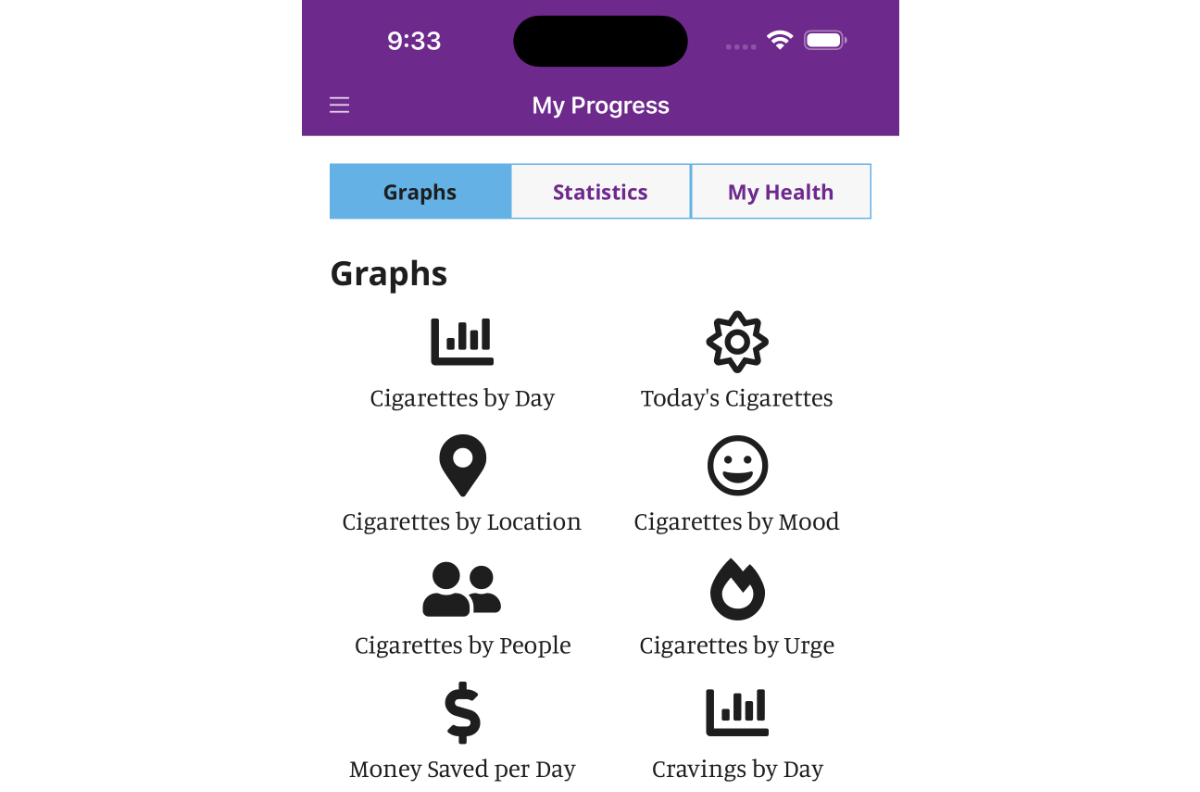
"My progress" page in the My Change Plan-Women app.

We co-developed gender-informed content consistent with the best available evidence regarding women-centered care [17,18]. The content is anchored within gender and sociopharmacology theories [10,19]. As such, the content refrains from using harmful gender norms to promote cessation, such as promoting quitting by emphasizing the ill effects of smoking on women’s appearance, rather than on women’s general health. Also, the content refrains from coercion to quit through shaming or blaming women for their smoking or inability to quit. Instead, it respects the autonomy of women to make changes when they are ready, accepting harm-reduction goals such as a gradual reduction rather than a complete quit. One way to achieve this is by placing an emphasis on preparations for quitting (eg, tracking the number of daily cigarettes (Figure 1-3), delaying the first cigarette of the day). Also, the content encourages the acceptance that quitting is a journey and that for some, it may take months or even years to achieve. Consistent with the sociopharmacology and gender theories, the content provides examples of how adverse social circumstances (eg, intimate partner violence, lone parenting) make nicotine particularly rewarding and that stress related to these adverse social circumstances then becomes a conditioned cue for cigarette smoking [10]. It then provides suggestions from other women on how to overcome these challenges such as identifying available supports in one’s environment, using smoking cessation medications, and finding alternative healthy behaviors for coping with stress.

**Figure 4:**
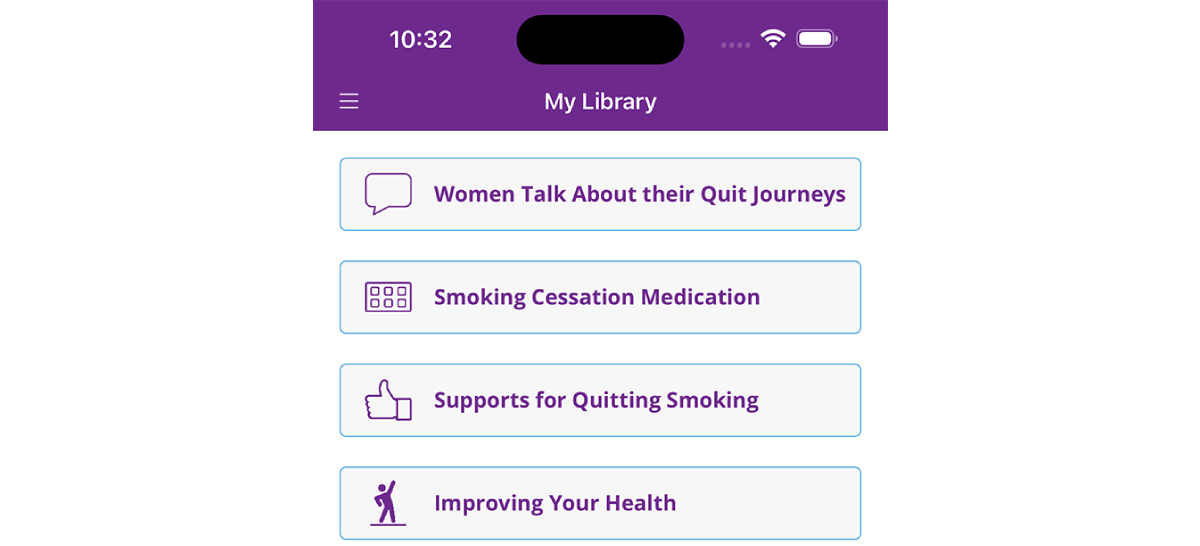
"My library" in the My Change Plan-Women app.

**Figure 5:**
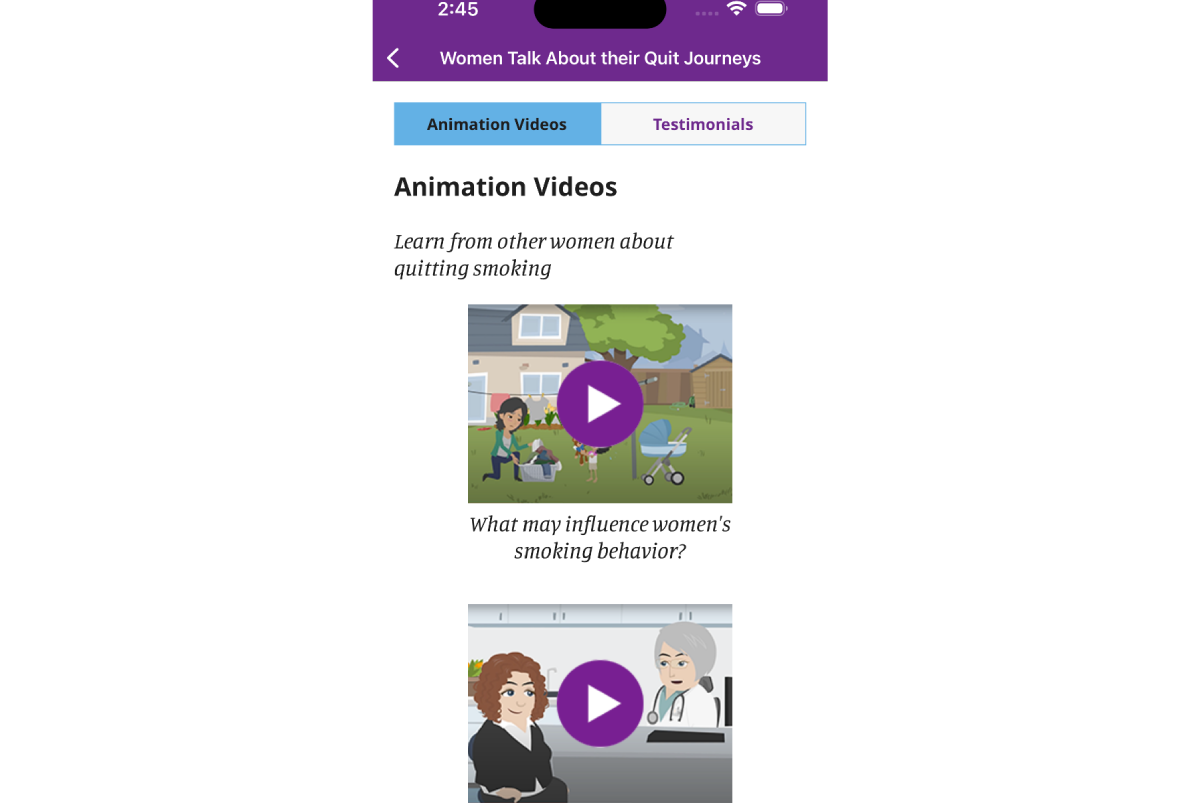
Animation videos in the My Change Plan-Women app.

**Figure 6:**
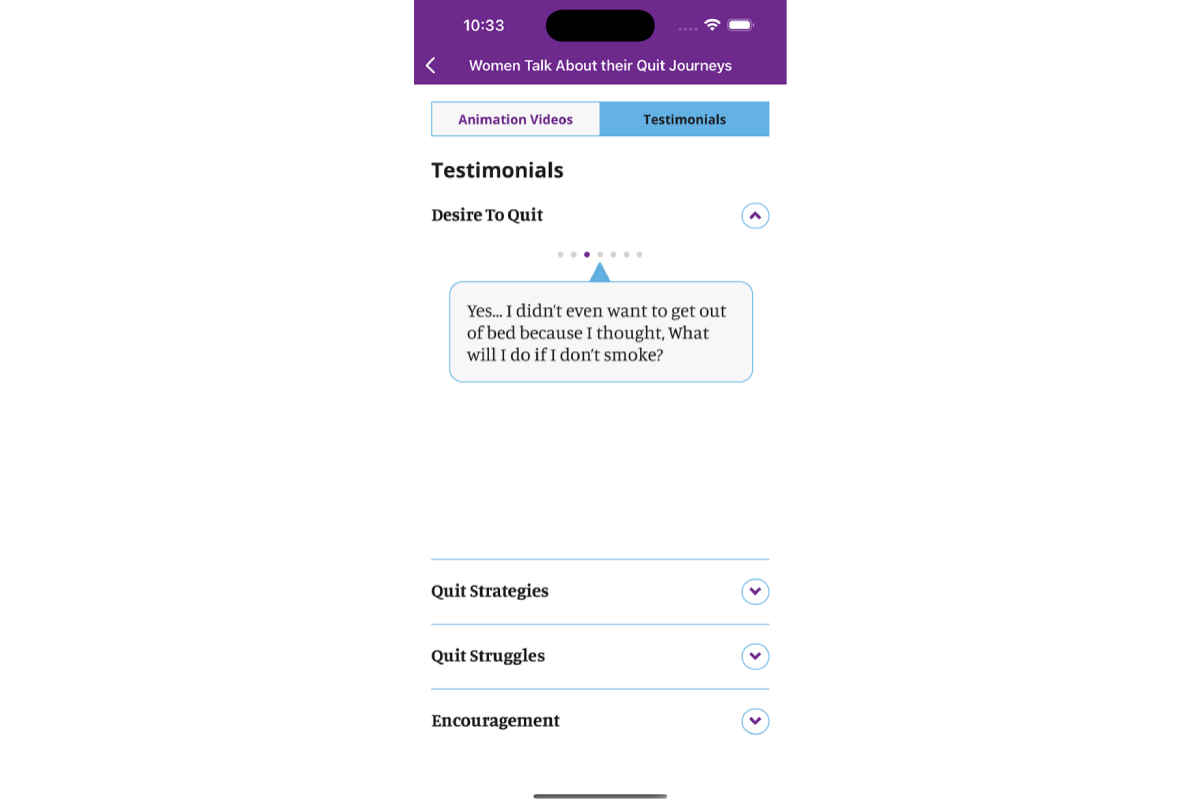
Testimonials in the My Change Plan-Women app.

We included the newly developed content in the app’s library under a tab titled “Women Talk about their Quit Journey” (Figure 4-6). This tab includes written information in the form of testimonials that bring forward the voices of other women offering strategies for quitting and encouragement. Also, it includes 4 animation videos that summarize key concepts regarding women-specific barriers to quitting and how to overcome them in a user-friendly manner. The app includes the following four videos, for which full transcripts are included in Multimedia Appendix 1:

1. A woman’s monologue: a woman talks about her life-long relationship with cigarettes, from initiation, through maintenance and the decision to quit, highlighting gender-specific motives for smoking and barriers to quitting.
2. Smoking and Stress: a dialogue between a woman and her physician regarding the link between smoking and stress.
3. Women’s dialogue: a group of 3 women talks about their experiences of smoking, their challenges in quitting, and what helped them overcome the challenges.
4. Initiating change to my smoking: a woman describes her first steps in the smoking cessation journey.

### Study Design, Setting, and Participants

This study is a prospective, single-group, mixed methods investigation to test the acceptability and feasibility of delivering a gender-specific smoking cessation intervention through a smartphone app. The study is being conducted in the INTREPID lab at the Centre for Addiction and Mental Health (CAMH) in Toronto, Ontario. We aim to recruit 30 women who smoke to test the MCP-W app.

### Recruitment and Procedure

We will promote the study among primary care providers who deliver the STOP smoking cessation program in Ontario, and who will be asked to refer interested patients. We will place recruitment flyers in our hospital (CAMH) bulletin boards, so interested women will be able to self-refer. In addition, we will reach out to previous STOP program participants who have expressed interest in participating in research studies. Interested patients will be screened against eligibility criteria. Those deemed eligible will be offered to enroll in the study after providing written informed consent.

Participants will be granted access to use the MCP-W app on their personal smartphone devices for a trial period of 28 days and provided a short demonstration of the app and its features. We have both Android and Apple versions of the app. During the trial period, participants will be asked to complete surveys at 2 time points: baseline, and at the end of the trial period (Multimedia Appendices 2-4). Once the 28-day trial period concludes, a research analyst will reach out to the participants and schedule a semistructured interview (Multimedia Appendix 5). During the interview, participants can provide additional feedback regarding their experience with the app and offer suggestions for improvement. The total duration of participant involvement in the study will span between 5 and 8 weeks. All interactions with the participants will be conducted remotely using the secure videoconferencing software, Webex, or via telephone. Quantitative and qualitative data analyses will be done within 3 months after the 28-day trial period concludes. Inclusion and exclusion criteria are shown in Textbox 1.

**Inclusion and exclusion criteria**

**Inclusion criteria**

- Self-identify as a woman
- Ages of 25-65 years
- Smoke at least 5 tobacco cigarettes per day
- Own a smartphone and have continuous internet access
- Have experience with using smartphone apps (ie, Facebook, Weather, News)

**Exclusion criteria**

- Does not feel comfortable using smartphone apps
- Severe general health or mental health conditions that interfere with study compliance
- Pregnancy, breastfeeding, or trying to become pregnant in the next 3 months

### Outcome Measures

#### Primary Outcome

The primary outcome of this study is the acceptability of the intervention. To define acceptability, we will use the Theoretical Framework of Acceptability (TFA) [24], which assesses the extent to which the app is perceived to have achieved its intended purpose. Participants will rate their perception of the app's effectiveness using a 5-point Likert scale (Multimedia Appendix 3), ranging from “strongly disagree” to “strongly agree,” in response to the statement: “Using the app is likely to help me make changes to my smoking habits.” Acceptability will be assessed using the surveys disseminated to participants at baseline and at the end of the 28-day trial period. To assess the extent to which participants engage with the app, our second primary outcome is feasibility, as evidenced by the number of days participants actively used (ie, logged into) the app during the 28-day trial period.

#### Secondary Outcome

The secondary outcome is the participants’ motivation to change their smoking behavior. Motivation to change will be assessed through 3 validated questions that rate reasons, taking steps, and ability to change one’s smoking [25]; the questions ask for ratings for the following statements: (1) it is important for me to change my smoking habits (reduce or quit), (2) I am trying to change my smoking habits (reduce or quit), and (3) I could change my smoking habits (reduce or quit). Each item will be rated on a Likert scale ranging from 1 to 5, with the following categories under the numbers: definitely not (1), probably not (2), maybe (3), probably (4), and definitely (5).

#### Tertiary Outcome

The tertiary outcome will be participants’ smoking habits as evidenced by the number of daily cigarettes [26] (Multimedia Appendix 3). We will measure any changes to participant smoking habits from baseline to end of trial. In addition, participants will report on the number of deliberate quit attempts (24-hour smoke-free period) in the preceding week both at baseline and at end of trial.

#### Exploratory Outcomes

Following the 28-day trial period, at the final survey we will assess participant perceptions on the usability of the app using the System Usability Scale [27] (Multimedia Appendix 4). This scale is a validated questionnaire to assess participants’ experiences regarding the ease of use of a new website or app platform.

### Demographic Variables

Demographic information will be collected from participants using a baseline survey at the start of the 28-day trial period (Multimedia Appendix 2). Demographic information collected on the survey will include age, gender identity, education level, marital status, household income, general health, and medical comorbidities.

### Data Analysis Plan

#### Sample Size Calculation

This is a single-arm study testing the acceptability and feasibility rather than the effectiveness of the MCP-W app in supporting change in smoking behaviors. The literature suggests a sample size of 30 participants for feasibility and usability studies [28].

#### Primary Outcomes

The primary outcome is the acceptability and feasibility of the MCP-W app.

We will measure acceptability by calculating the proportion of participants responding “agree” or “strongly agree” to the statement, “using the app is likely to help me make changes to my smoking habits.” We will deem the MCP-W app as acceptable if this proportion is equal or greater than 50% (15/30); needs further work if it is 26% (8/30) - 49% (14/30); and to be unacceptable if this proportion is 25% (7/30) or lower. As the small sample makes multiple imputations impractical, we will impute nonresponse values at the final follow-up to “do not agree,” on grounds that low study engagement is likely to indicate lower acceptability of the intervention.

For feasibility, we will calculate the proportion of participants who used the app on at least 7 days out of the 28-day trial period. We will use the same thresholds (50%+; 26%-49%; and 0%-25%) for interpreting this outcome. As these data are based on Google Analytics, they will be available for all participants; no missing data should occur. We are able to access Google Analytics data through Google Firebase. Firebase is a set of backend cloud computing services and application development platforms provided by Google. We will assign unique login IDs for each participant. Every time a participant opens the app and uses their unique ID to login, the data is recorded. This will allow us to observe how many days the app was used by each research participant. We do not expect to have any missing data on feasibility measures as they are automatically collected on the backend of the app (ie, the app opened vs not opened).

To understand the precision of our estimates, we will calculate the exact binomial 95% CI. However, our decisions will rest on the point estimates only.

#### Secondary and Tertiary Outcomes

The secondary outcome will assess participant’ motivation to change their smoking behavior. The tertiary outcome will be changed in the participant’s smoking behavior. We will report all secondary and tertiary outcomes descriptively at all-time points at which data are collected. We will also calculate the proportions showing increased motivation, substantial reductions (≥50%) in cigarettes per day, and an increased number of quit attempts. Although this study is not powered to detect significant differences, we will calculate CI and perform statistical tests to gain insight into the precision of results. We will use McNemar tests for binary outcomes; exact Wilcoxon signed-rank tests for paired ordinal data; and linear, Poisson, or negative binomial regression for cigarettes per day, depending on the observed distribution.

If unanticipated issues necessitate any deviation from these approaches, we will describe the analysis originally intended, the analysis actually used, and detail the reasons for differences.

We will use Stata (version 16; StataCorp LLC) to conduct the data analyses.

#### Qualitative Analysis

To analyze the data from the semistructured interviews, we will employ a deductive (top-down) approach. The coding process will be guided by the 7 constructs of the TFA [24] to assess the user experience with the app: affective attitude, burden, perceived effectiveness, opportunity costs, intervention coherence, self-efficacy, and ethicality. Analyzing the qualitative data using this method will allow for a rich description of how participants perceived the acceptability of the women-informed app. Researchers with appropriate training will thoroughly review the data and assign relevant excerpts to the respective codes. Through these analyses, we anticipate that our codes will closely align with the initial codebook we established. Researchers will meet regularly during the coding process and any disagreements will be resolved through consensus.

### Ethical Considerations

The study was reviewed and approved by the institutional Research Ethics Board of CAMH in Toronto, Ontario (protocol 122-2023). Participants will receive CAD $30 (US $21.59) in compensation at the time of baseline survey completion, and an additional CAD $30 (US $21.59) following the completion of the posttrial interview.

## Results

We secured funding for the study in July 2022 and completed the development of the MCP-W app in September 2023. Participant recruitment for testing of the app commenced in February 2024 and is expected to be completed by December 2024. We will analyze the data upon completion of data collection from all 30 participants. We expect to share the results of this acceptability trial in the middle of 2025.

## Discussion

### Principal Findings

This study will examine whether women who smoke find the MCP-W app acceptable and feasible for their smoking cessation needs. We will also explore whether using the app is associated with changes in the motivation of women to quit or reduce smoking, the number of cigarettes smoked per day, and the number of smoke-free days in the last 7 days. We will be able to expand our understanding of the quantitative findings using rich qualitative data captured in the exit interviews.

### Novelty of the Study

Smartphone apps are increasingly used to improve health behaviors among the general public. However, they are seldom customized based on the gender of the user. This may pose a disadvantage for health behaviors that are directly influenced by the individual’s gender, such as in the case of tobacco smoking. Recently, a number of studies attempted to tailor smoking cessation apps according to gender, including an app to support women in making a change to multiple health behaviors [29], and an app to support women who are pregnant [30]. This study adds to the scholarly work in this domain by developing a smoking cessation app with gender-specific content and assessing its acceptability and feasibility.

### Strengths and Limitations

This study is a novel attempt to deliver a gender-informed intervention for smoking cessation anchored within gender theory. Given the wide reach of smartphone apps and the relative ease of customizing content to specific population groups, it has the potential to improve women’s health. Since the app was codeveloped with patient partners, it's expected to be both relevant and acceptable to women who smoke. The development of MCP-W leveraged existing assets, such as MCP, the gender-neutral smoking cessation app. This not only reduces development costs but also provides a conceptual model for tailoring content to other groups facing disparities in smoking quit rates.

This study will provide us with information on the acceptability and feasibility of the gender-informed app but due to a small sample size, it is underpowered to study the app’s effectiveness in supporting smoking cessation. Furthermore, the majority of participants recruited for testing the app are individuals seeking treatment for smoking, which could potentially limit the generalizability of the app’s acceptability among women with lower interest in quitting.

### Future Directions

The findings from this study and the feedback provided by research participants will inform the development of additional gender-specific digital tools including a gender-specific smoking cessation conversational agent (ie, a chatbot). In addition, adding a gamification component to the current MCP-W app may increase its acceptability and be an avenue for future research [29,30].

### Knowledge Dissemination Plan

We plan to publish the results in a scientific publication and present the results at scientific conferences, namely, those of the North American Primary Care Research Group, and the Society for the Research of Nicotine and Tobacco. Also, we plan to share the results with the Gender, Evidence, and Health Network, which promotes gender equity in scientific research.

## Appendix

Multimedia Appendix 1 Video scripts.

Multimedia Appendix 2 Baseline demographic survey instrument.

Multimedia Appendix 3 Surveys for baseline and follow up.

Multimedia Appendix 4 System Usability Scale survey instrument for the follow up survey.

Multimedia Appendix 5 Interview guide.

## Abbreviations

CAMH: Centre for Addiction and Mental Health
MCP: My Change Plan
MCP-W: My Change Plan-Women
STOP: Smoking Treatment for Ontario Patients
TFA: Theoretical Framework of Acceptability

## Data Availability

The data sets generated during and/or analyzed during this study are not publicly available due to privacy limitations set forth by our institution. The corresponding author will consider sharing data provided the researchers obtain approval from their institutional review board for their suggested data analysis plan.
