# Supplementary material for: A Gender-Informed Smoking Cessation App for Women: Protocol for an Acceptability and Feasibility Study
Source: JMIR Res Protoc. 2024 Dec 10;13:e60677. doi: 10.2196/60677 (PMC11668997; doi:10.2196/60677)
Supplement: Multimedia Appendix 1 [file resprot_v13i1e60677_app1.docx]

**Video Script #1:** [https://youtu.be/5c4k-TIoKQg](https://urldefense.com/v3/__https:/youtu.be/5c4k-TIoKQg__;!!FxkXuJIC!ZdODa5cMQxkw12ZCPEkJtHfxGOsi3zjaxUA-Eu2rCrZ5P5uMVZes8a6yu0lprpvKilcM8yva6YXMu4wmASvLJiaR$)

**Purpose**:
To increase awareness and knowledge about the role smoking plays in women’s lives.

To provide information on the interplay between gender and smoking and provoke thoughts on the meaning of smoking for each individual woman

**Learning Objectives**

After watching the videos, the audience will be able to:

- Describe specific roles that cigarettes play in women’s lives
- Appreciate the challenges in finding a replacement for cigarettes given their many roles when planning for a quit

**Setting:** Women talk about the role cigarettes play in their lives

**Character Description:**

- Mandy.
- 50-year-old woman, South Asian, married with adult children.
- Works as an administrative assistant.
- Contemplating making a change to her smoking behaviour for her 50^th^ birthday.

**Background on Scenario**

Mandy is a 50yo woman that works as an administrative assistant. She is invited for an interview to talk about her relationship with cigarettes over the years as she is thinking about making a change to her smoking.

**Animation Script**

Prompt (whole screen caption): What influenced you to start smoking when you were younger?

Mandy: I started smoking in my last year of high school, I was 18 at the time. It seemed to me then as something “cool” kids did. It helped me feel connected to some of the other kids who smoked. (video in the background). I also knew that my parents disapproved of it, so it made me feel like I was rebelling against them; being modern as opposed to being “old fashioned” like my parents were, where women did not smoke. (video in the background).

Prompt (whole screen caption): How were cigarettes part of your life later on?

Mandy: Well, I got married rather young and by the time I was 25, I was already a mother of two. My husband and I were barely making ends meet at the time. He was working long hours mostly outside the home and I was juggling between my day job and caring for our two young children. (video in the background) Those years were very stressful. I turned to smoking to deal with the stress of day-to-day demands. When things were too much to handle, I could always distract myself by lighting up a cigarette. Those were the only few minutes in my busy day that I was by myself, relaxing, not having to think about the kids, the house chores, my job (video). I guess ever since then cigarettes are my “go to” when it comes to dealing with stressful situations and taking time to myself.

Prompt (whole screen caption): Does smoking relate to certain environments or situations in our life?

Mandy: mmmmm, that’s a good question. Yes. I think so. For example, when my children were little, my husband and I would argue a lot. At times, the arguments would get heated (video) which would make me feel lonely and scared. I remember I used cigarettes as an excuse to “step out” of a difficult conversation. Cigarettes helped me take control over the situation, made me feel safer, calmer.

…. another thing that comes to mind is the exact opposite, are also a great tool to bring people together. In fact, one of my best friends, a colleague of mine, she and I are both administrative assistants at the office. We met 10 years ago in an alleyway outside the office, where we both smoked. We’ve been close ever since. We meet every day outside the office for a coffee and cigarette right after lunch (video). We share this special bond over smoking, many of the other workers disapprove of our smoking, but we have our cigarettes and we have each other.

Prompt (whole screen caption): What do you think about cigarette smoking these days?

Mandy: Well, I am aware that smoking cigarettes is not good for you. I am 50 now and so far, I’ve been pretty healthy but I am starting to cough up in the mornings. When I went to see my doctor for the cough, she told me I should quit smoking. <sigh> I find that quitting is hard. I wasn’t successful in quitting even though I tried a few times. Besides, cigarettes and me go back a while back. I’ve been smoking since I was 18. Cigarettes were with me through good times and bad, they’ve been something constant, something I could rely on. You may even call it a “best friend”. It feels difficult to let them go.

Prompt (whole screen caption): What would your life have to be like in order for you to not smoke?

Mandy: mmmmm…. it’s hard to imagine a life without cigarettes since I’ve been a smoker for so long. If I look closely at my life, I see that smoking is built into my daily routine; smoking in my work breaks with my best friend (pop up video balloon- friend); smoking cigarettes as a way to manage conflict and deal with stress (pop up video balloon- husband); and smoking is something I do when I want to take time just for myself (pop up video balloon- alone time).

I see that smoking plays many different roles in my life. If I wanted not to smoke, I’d have to find other ways to deal with stress, other ways to break the routine in order to keep to myself, other ways to interact with my best friend without smoking. I think it may take some time to make these changes but I would like to try, my health is what matters.

**End of Video Script #1**

**Video Script #2**

**Purpose**: To increase awareness and knowledge about how cigarettes contribute to stress rather than alleviate it.

**Guiding principles:**

- Psychoeducation on the links between smoking and stress
- Smoking/nicotine gives a time-limited pleasurable effect
- Nicotine w/d causes the opposite
- Smoking mostly relieves “nicotine stress” and not real stress

**Learning Objectives for Simulation Component**

After watching the videos, the audience will be able to:

- Identify how smoking can both reduce stress (Short term) and increase stress (long term)

**Character Description:**

- Sofia, 30yo woman.
- She is working towards becoming a registered real estate professional.
- Meeting with her family physician, a 60yo woman.

**Background on Scenario**

Sofia shares her experience of starting to smoke occasionally at social settings but how smoking has escalated over her 20s and now she finds she smokes daily to manage stress related to her work.

**Animation Script**

Sofia: Hi Doctor, I’m thinking about quitting smoking, but not sure I can manage my stress without cigarettes. You see, I’ll be taking one of the real estate licensing exams in the fall, and I’m quite nervous.

**Doctor:** I see. You feel cigarettes help you deal with stress. Would you like to learn more about cigarettes and stress?

Sofia: Yes, sure.

**Doctor**: Ok, good. Can you tell me about your experience with cigarettes?

Sofia: I started smoking when I was 25. I was in college then and I remember feeling anxious about meeting new people. When I tried smoking, it helped me relax. In the past few months, I’m studying for my exams, it is really stressing me out, so I’m noticing I’m smoking more and more.

**Doctor**: Yes, many young people start to smoke when they are in environments where others smoke. The nicotine we inhale in cigarette smoke releases dopamine in the brain which makes us feel happy and relaxed.

Sofia: So, it’s true that cigarettes can help reduce stress, then?

**Doctor**: The story is a bit more complicated. Nicotine does give us a temporary “feel good” experience, but it does not last for long. After we finish smoking a cigarette, nicotine slowly leaves our body and we then go into a state of nicotine withdrawal. This is also called “nicotine stress” causing us to feel restless, irritable, moody and anxious which is the opposite of stress reduction. The next time we smoke, we fix the state of nicotine withdrawal by inhaling more smoke with nicotine into our bodies. That’s why we feel cigarettes relieve our stress.

Sofia: so, you mean that the cigarettes relieve the stress of nicotine withdrawal? Is that why I feel relaxed after I smoke my first cigarettes in the morning?

**Doctor**: Exactly. The levels of nicotine go up in our body when we smoke. We have a temporary relief from nicotine w/d when we smoke but it is short-lived. If we do not smoke, again we will go into w/d again and re-experience “nicotine stress” just to be relieved by the next cigarettes we smoke and so forth.

Sofia: Yes, I see. It seems nicotine keeps me in the vicious cycle of feeling relieved temporarily only to feel stressed again and smoke again.

**Doctor:** Right. It can be a challenging to break this cycle on your own.

Sofia: Is there anything that can help with nicotine w/d? something to help break this cycle?

**Doctor:** Yes. In fact, all of the medications we use for smoking cessation are effective because they do just that; reduce the intensity of nicotine withdrawal a person feels when they stop or reduce their smoking. Using medication helps people go longer without smoking, cut down and eventually quit. Counselling can also help you break this cycle by giving you skills and strategies to deal with nicotine cravings/urges and other signs of nicotine w/d.

Sofia: That sounds good. Thanks doctor, I would be interested in learning more about these options.

**End of Video Script #2**

**Video Script #3**

**Purpose:** To learn about the quit smoking journey as experienced by women

**Learning Objectives for Simulation Component**

After watching the videos, the audience will be able to:

- Describe challenges and opportunities for changing smoking behaviour:

-what things make quitting hard?

-healthy alternative to dealing with stress

-Who might support you in quitting smoking?

-reduction of exposures to others’ smoking

-Where to start my quit journey?

- words of encouragement for quitting from PWLE

**Setting:** Women talk about their quit journey

**Characters:**

Lynn - 45 yo South Asian woman,

Stephanie - 55yo Caucasian woman, and

Nora - 35yo Asian woman

**Animation Script**

- **Could you tell me about challenges you had in your quit smoking journey? (i.e, what made it hard for you to quit?)**

**Life Long Habit** (Lynn) : “Just the amount of time I have been a smoker at this point makes it harder as smoking has really become ingrained in my everyday routine”

**Lack of practical support** (Stephanie):“Doctors tell you, you should quit, but they don't give you a path or suggestions. Everyone in the room agrees I should quit, but how???

**Close relationship with cigarettes** (Stephanie): “I had a reliable relationship with my cigarettes. Cigarettes were my best friend and sometimes my only friend, I really didn't want to quit. I was kind of ambivalent.”

**Dealing with stress** (Lynn):~having to deal with other important things in my life like not having a place to live in or having financial struggles did not allow me to focus on quitting~

- **People cite “coping with stress” as one of the main reasons for smoking. What are some healthy ways you found helpful when dealing with stress?**

**Talking to others** (Lynn): “Some ways I have found to cope with stress is to really talk through stressful times and emotions...to reach out to my counsellor, a friend, my Mom...someone who can help me see that the extreme feelings are not going to last forever”

**Mindfulness and Breathing** (Stephanie): “Learning to acknowledge to myself when I am feeling overwhelmed… and then quieting my mind… I tell myself to stop and take 3 deep belly breaths”

**Keeping busy with leisure activities** (Nora):“I have a dog and I play with him, it’s a real form of joy… I also exercise… that helps me destress”

**Dealing with other addictions** (Lynn) : “Being …sober for 5+ years has helped me because the chaos and the stress of being underhoused and in extreme poverty has become non-existent for me and this reduces my stress. Also going for a walk or doing a Youtube workout video at home definitely helps to cope with a stressful moment”

- **Did you have support from others in your quit? Was it important?**

**Support from family and friends** (Lynn): “My Family is supportive and not pushy about the quitting process and my friends are mindful to try and not smoke around me when I am trying a period of abstinence”

**Support from other ex-smokers** (Stephanie):“People who have never smoked, don't get how hard quitting is…. it's easier with a smoker because they know how hard it is.”

**Support from other women ex-smokers** (Nora):“I found it more helpful to talk to other smokers and especially, other women smokers who quit… they would relate to what you are going through, that was key”

**Group support** (Stephanie): “I got referred to some group by my doctor and to see those people and everybody's success and failures - all of those things are important to me and they really helped me

**Apps for smoking** (Nora): “I got connected with a text-based app about smoking. And so, every morning I'd get a text and that was also kind of good”

- **Exposure to smoke in the environment. What can we do if we want to change our smoking but others (i.e. family, friends, colleagues) around us smoke?**

**Avoid spending time outside with other smokers** (Lynn): “Stop hanging out for a little while if necessary”

(Stephanie): “The only place cigarettes come into my life is at work. I'd rather do something else (indoors) probably keep working …than go out and stand with the other smokers”

(Nora): “I still don’t go outside with friends who smoke”

**Letting others know** (Lynn):“Share that you want to quit smoking and you will need support. Asking if your activities together not include smoking anymore”

- **Could you recommend some first steps (harm reduction) for women who are hesitant about quitting at this time?**

**Keeping track of your smoking** (Lynn): “For me what has been really helpful is tracking my smoking intake to both see how much I am smoking and how much I am spending”

**Cut down gradually** (Nora): “every week try to reduce by 1 cigarette”

**Change your surroundings** (Nora): “Changing environments has helped me immensely. Remove the smoking chair, make small tweaks to your life like throw the ash tray. Getting rid of anything that you associate with smoking”

- **What does it take to be successful in quitting? Any words of encouragement/tips you can share with other women?**

**Be patient, quitting takes time**

(Lynn): “Realize how long smoking has been a part of your life and that it will take time, commitment and dedication to also have it not be part of your life anymore”

(Lynn): “NEVER GIVE UP just keep going despite setbacks”

(Nora): “It might take you many tries but eventually it might work. It is hard but don’t stop trying ever.”

**Look for support** (Stephanie): “Have someone in your corner that you can talk to when you want to smoke”

**End of Video Script #3**

**Video Script #4**

**Purpose**:
To increase awareness and knowledge about how to start the smoking cessation/reduction journey

Guiding principles:

- Non-judgmental empowering approach that focuses on women’s health
- Women’s autonomy is respected to choose goal for smoking (reduction, cessation, other)
- Contextual to women’s life circumstances including intersectionality with socioeconomic status, ethnicity, mental illness/addictions, adverse childhood events or traumatic events.

**Learning Objectives for Simulation Component**

After watching the videos, the audience will be able to:

- Identify opportunities for change in smoking including:

-gradual reduction

-reduction of exposures to others’ smoking

-identifying supports for quitting

-improving other aspects of health

**Setting:** Women talking about initiating change in their smoking

**Characters:**

- Mandy (Age 49, South Asian background) and Lisa (Age 45, White) are co-workers (administrative assistants) and good friends.
- They usually meet for their lunch break to smoke and have a coffee together.
- Mandy will be turning 50 soon and is looking to improve her health by making a change to her smoking.
- She brings this up for discussion with her friend, Lisa, during their lunch break. Through this discussion, they identify opportunities for change.

**Animation Script**

Lisa: Hi Mandy, it’s so nice to see you today, how are you doing? *(meeting in an alley, Lisa is pulling out her cigarettes pack, Mandy is joining)*

Mandy: Great to see you too, Lisa. I’m doing well. <pause> You know Lisa, I’m turning 50 in a few months and I’m trying to improve my health. My doctor and I had a conversation about it. She said that the #1 thing I could do is to quit smoking.

Lisa: Yeah, Yeah, Yeah, I’m sure your doctor is just like everyone else, telling you need to quit. We’ve heard it all before.

Mandy: I know what you mean, Lisa, but our conversation was different. My doctor wanted to understand how cigarettes fit into my life. I told her that many people I know smoke and that I’ve been a smoker almost my entire life.

Lisa: For sure, smoking is a habit, it's part of our routine.

Mandy: Right. I feel the same way. I really enjoy our cigarette breaks together. It’s something I look forward to every morning when I come into work.

Lisa: Me too! We’ve been doing this together for …mmmm… 10 years now! (smiling)

Mandy: There are other cigarettes that will be hard to let go of, like the ones I smoke when I’m feeling stressed for example, or those that I have after dinner, that allow me just a few moments of peace and quiet to myself.

Lisa: Yes, I hear you.

Mandy: I’ve always felt quitting is too hard, but my doctor came up with some suggestions that I liked. I realized, I do not have to give up cigarettes all at once, but can plan to make changes over time and at my own pace.

Lisa: That makes sense, who hasn’t tried the “cold turkey” quit? It never worked for me. Taking baby steps seems logical. What’s your plan?

Mandy: Lisa, you are a dear friend, so that’s why I’m talking to you first. I wanted to ask how you would feel about changing our lunch break routine?

Lisa: Change? In what way? (looking surprised)

Mandy: I still want to meet you every day at lunchtime but I want to try not to smoke.

Lisa: You mean you are planning to quit?

Mandy: Quitting seems too hard for me at this time, but I thought I would try to cut down on my smoking. I smoke about 10 cigarettes every day, and 2 cigarettes come in at lunchtime. I’d like to try and cut those out of my routine.

Lisa: Good for you, Mandy. I’m not ready to give up on my cigarettes just yet. So, how will our lunch break look?

Mandy: I’ve been thinking, we could stay in the office’s kitchenette and prepare a healthy lunch, cut up some veggies and make our own salad. That way, if I stay indoors, I will not be tempted to smoke, but I can still spend some time with you and if you wanted to smoke, you would go outside without me.

Lisa: Yeah, Mandy, I would like that. I guess we could both eat better we usually just settle for the coffee and the cigarettes.

Mandy: Thanks, Lisa. Your support means a lot to me. Making this change by myself would be really hard. (Hugs)

Lisa: You can count on me, Mandy! Good for you for starting to think about your smoking, I will also have to do something about it…eventually…

**End of Video Script #4**
