# Supplementary material for: A Gender-Informed Smoking Cessation App for Women: Protocol for an Acceptability and Feasibility Study
Source: JMIR Res Protoc. 2024 Dec 10;13:e60677. doi: 10.2196/60677 (PMC11668997; doi:10.2196/60677)
Supplement: Multimedia Appendix 3 [file resprot_v13i1e60677_app3.docx]

1. Perceived effectiveness:

Please indicate how much you agree or disagree with the following statement:

"Using the app is likely to help me make changes to my smoking habits”.

Strongly disagree (1), Disagree (2), Neutral (3), Agree (4), Strongly Agree (5)

1. Smoking behavior:

How many cigarettes per day do you smoke?

1. Quit attempts:

How many days in the past seven days were smoke-free 0 days (i.e. being smoke free for 24 hours)?

1. What do you think about changing your smoking habits?
   1. It is important for me to change my smoking habits (reduce or quit)
      Definitely Not (1), Probably Not (2), Maybe (3), Probably (4), and Definitely (5).
   2. I am trying to change my smoking habits (reduce or quit)
      Definitely Not (1), Probably Not (2), Maybe (3), Probably (4), and Definitely (5).
   3. I could change my smoking habits (reduce or quit)

Definitely Not (1), Probably Not (2), Maybe (3), Probably (4), and Definitely (5).
