# Supplementary material for: A Gender-Informed Smoking Cessation App for Women: Protocol for an Acceptability and Feasibility Study
Source: JMIR Res Protoc. 2024 Dec 10;13:e60677. doi: 10.2196/60677 (PMC11668997; doi:10.2196/60677)
Supplement: Multimedia Appendix 4 [file resprot_v13i1e60677_app4.docx]

This is a standard questionnaire that measures the overall usability of a system.  Please select the answer that best expresses how you feel about each statement after using the app.

|  | Strongly Disagree | Somewhat Disagree | Neutral | Somewhat Agree | Strongly Agree |
| --- | --- | --- | --- | --- | --- |
| 1. I think I would like to use this app frequently. |  |  |  |  |  |
| 1. I found the app unnecessarily complex. |  |  |  |  |  |
| 1. I thought the app was easy to use. |  |  |  |  |  |
| 1. I think that I would need the support of a technical person to be able to use this app. |  |  |  |  |  |
| 1. I found the various functions in this app were well integrated. |  |  |  |  |  |
| 1. I thought there was too much inconsistency in this app. |  |  |  |  |  |
| 1. I would imagine that most people would learn to use this app very quickly. |  |  |  |  |  |
| 1. I found the app very cumbersome to use. |  |  |  |  |  |
| 1. I felt very confident using the app. |  |  |  |  |  |
| 1. I needed to learn a lot of things before I could get going with this app. |  |  |  |  |  |

How likely are you to recommend this app to others? (Please circle your answer)

Not at all likely    0         1         2         3          4          5          6          7          8           9          10    Extremely likely
