# Supplementary material for: A Gender-Informed Smoking Cessation App for Women: Protocol for an Acceptability and Feasibility Study
Source: JMIR Res Protoc. 2024 Dec 10;13:e60677. doi: 10.2196/60677 (PMC11668997; doi:10.2196/60677)
Supplement: Multimedia Appendix 5 [file resprot_v13i1e60677_app5.docx]

*Good morning/afternoon. My name is [Research staff name], my pronouns are _____ and I work at CAMH. As you might recall on (date consent was signed), you agreed to participate in this interview. For this study, we are interested in hearing about your experience using the smoking cessation app called “My Change Plan - Women” for the last 28 days. I will also ask you a few questions about your smoking behaviours and reasons for quitting*

*I will be audio recording the session because we don't want to miss any of your comments. People often say a lot of helpful things in these discussions, and we can't write fast enough to get them all down. We expect this interview to last no longer than60 minutes. We are interested in hearing your views and appreciate your opinions. There are no right or wrong answers.*

*We will be on a first-name basis today, but we won't use any names in our reports. Please be assured that whatever we discuss today is completely confidential. We will use the information that you share with us today to help us adjust the features of the app to improve its use for other women who smoke.*

*I would like to remind you that there are no known risks associated with taking part in this interview. Please note that during the interview there are some questions that ask you to reflect on your smoking behaviours. These questions may cause you to feel uncomfortable, or recall difficult memories. You can skip questions you do not want to answer. You can take breaks if you would like to. You can also tell the interviewer immediately if you feel uncomfortable or upset and end the interview at any time.*

*Before we begin, do you have any questions?*

*Do you agree with continuing with the interview? Do you give your consent to having the interview audio recorded?*

*Well, let's begin.*

| Theoretical Framework of Acceptability (TFA) construct | Interview Questions |
| --- | --- |
| 1) **Affective Attitude**- How an individual feels about MCP-W? | Is there anything in particular that *you really liked* about the MCP-W app? What features in the app stood out for you?  Is there anything in particular that *you did not like* about the MCP-W app?  Please share your thoughts on what you liked about the MCP-W app.  Probe: Can you tell me about any features that stood out for you?  Can you tell me about anything in particulate that you did not like about the MCP-W app? |
| 2) **Burden**- The perceived amount of effort that is required to use MCP-W | In your opinion, how easy or difficult *was it* to use the app? |
| 3) **Effectiveness**- The extent to which MCP-W  is perceived as likely to or has achieved its intended purpose | To what extent do you think the MCP-W *can help women make changes to their smoking?* Can you give examples of how the app can help? |
| 4) **Opportunity Costs** - The extent to which the benefits, profits or values must be given up to engage in MCP-W | Can you tell me about anything that you personally had to *give up* or had to  sacrifice in order to use the MCP-W app?  How difficult or easy was it to fit in using the app during a typical day? |
| 5) **Intervention Coherence**- The extent to which the participant understands MCP-W and how it works | To what extent do you think the MCP-W app focuses on topics that are important for women who smoke? |
| 6) **Self-Efficacy**- The participant’s confidence that they can perform the behaviours required to participate in MCP-W | Can you share how confident you felt in you ability to use the app and all of its features? |
| 7) **Ethicality**  The extent to which MCP-W has good fit with an individual’s value system | In your opinion, to what extent do you think the MCP-W *was* a good fit with your own values or beliefs? |
| 8) Suggestions | Can you share any suggestions on how to improve the app? |

1. What would your life have to be like in order for you to not smoke?

Can you tell me how the app has helped you take any steps towards quitting smoking if at all?

Probe: Can you tell us what those steps are?

1. The last question is seeking input from women who identify as women of color, indigenous or other visible minorities. Do you identify as belonging to any of these groups?

If yes- Tell me a bit more about the way you just identified yourself. Do you see any barriers to using this app by women of diverse backgrounds?

If no- Do you have any suggestions on how this app could better support women of diverse backgrounds?

*This brings us to the end of the interview. We asked you quite a few questions. Is there anything else that you would like to add that we missed?*

*Thank you for taking the time to answer our questions and sharing your feedback with us. This information will help us assess how acceptable the MCP-W app is for women who smoke, and ways in which we can improve the app for future use.*
